# First‐in‐Human Studies of MW01‐6‐189WH, a Brain‐Penetrant, Antineuroinflammatory Small‐Molecule Drug Candidate: Phase 1 Safety, Tolerability, Pharmacokinetic, and Pharmacodynamic Studies in Healthy Adult Volunteers

**DOI:** 10.1002/cpdd.795

**Published:** 2020-04-07

**Authors:** Linda J. Van Eldik, Lumy Sawaki, Karen Bowen, Daniel T. Laskowitz, Robert J. Noveck, Byron Hauser, Lynn Jordan, Tracy G. Spears, Huali Wu, Kevin Watt, Shruti Raja, Saktimayee M. Roy, D. Martin Watterson, Jeffrey T. Guptill

**Affiliations:** ^1^ Sanders‐Brown Center on Aging University of Kentucky Lexington Kentucky USA; ^2^ Department of Neuroscience University of Kentucky Lexington Kentucky USA; ^3^ Spinal Cord and Brain Injury Research Center University of Kentucky Lexington Kentucky USA; ^4^ Department of Physical Medicine & Rehabilitation University of Kentucky Lexington Kentucky USA; ^5^ Bluegrass Research Consultants, Inc. Versailles Kentucky USA; ^6^ Department of Neurology Duke University Durham North Carolina USA; ^7^ Duke Clinical Research Institute Durham North Carolina USA; ^8^ Duke Early Phase Clinical Research Unit Durham North Carolina USA; ^9^ Department of Pharmacology Northwestern University Chicago Illinois USA

**Keywords:** phase 1, cytokine, pharmacokinetics, pharmacodynamics, brain injury, neuroinflammation

## Abstract

MW01‐6‐189WH (MW189) is a novel central nervous system–penetrant small‐molecule drug candidate that selectively attenuates stressor‐induced proinflammatory cytokine overproduction and is efficacious in intracerebral hemorrhage and traumatic brain injury animal models. We report first‐in‐human, randomized, double‐blind, placebo‐controlled phase 1 studies to evaluate the safety, tolerability, and pharmacokinetics (PK) of single and multiple ascending intravenous doses of MW189 in healthy adult volunteers. MW189 was safe and well tolerated in single and multiple doses up to 0.25 mg/kg, with no clinically significant concerns. The most common drug‐related treatment‐emergent adverse event was infusion‐site reactions, likely related to drug solution acidity. No clinically concerning changes were seen in vital signs, electrocardiograms, physical or neurological examinations, or safety laboratory results. PK analysis showed dose‐proportional increases in plasma concentrations of MW189 after single or multiple doses, with approximately linear kinetics and no significant drug accumulation. Steady state was achieved by dose 3 for all dosing cohorts. A pilot pharmacodynamic study administering low‐dose endotoxin to induce a systemic inflammatory response was done to evaluate the effects of a single intravenous dose of MW189 on plasma cytokine levels. MW189 treatment resulted in lower levels of the proinflammatory cytokine TNF‐α and higher levels of the anti‐inflammatory cytokine IL‐10 compared with placebo treatment. The outcomes are consistent with the pharmacological mechanism of MW189. Overall, the safety profile, PK properties, and pharmacodynamic effect support further development of MW189 for patients with acute brain injury.

Acute brain injuries resulting from trauma or cerebrovascular injury, such as traumatic brain injury (TBI) and intracerebral hemorrhage (ICH), are major medical problems that cause considerable mortality and morbidity.^1^, ^2^, ^3^ In addition to damage from the initial insult, downstream pathophysiological events can result in further brain injury and lead to increased risk of longer‐term neurologic complications.^4^, ^5^, ^6^, ^7^, ^8^ A key downstream injury response occurring within the relevant injury‐to‐treatment time window is a dysregulated inflammatory response in the brain (neuroinflammation). A specific aspect of neuroinflammation, injury‐induced proinflammatory cytokine overproduction from abnormally activated glia, has been linked to subsequent neurological damage and cognitive deficits following acute brain injuries.^9^, ^10^, ^11^ In both animal models and human patients, acute brain injury induces a robust increase in cytokine levels in the brain that occurs in the first several hours to days after insult and then subsides. This acute proinflammatory cytokine surge is a key contributor to subsequent cerebral edema, long‐term neuronal dysfunction, and cognitive impairment. This mechanistic linkage and the attractive therapeutic time window of hours to days postinsult provide a rational therapeutic target for intervention in the acute care setting. Injury‐induced dysregulated glial activation is a critical convergence point for diverse central nervous system (CNS) insults that can compromise neuronal survival, and excessive glial activation is a major factor contributing to undesirable outcomes following acute brain injury. However, despite advances in our understanding of these cellular and molecular neuroinflammatory mechanisms underlying adverse neuronal sequelae following injury, approved therapeutics that target this pathological process are lacking. Although there have been significant advances in the medical management of patients with acute brain injuries, there is a clear and urgent need for interventions that improve neurologic recovery and outcomes.

To address this medical need, we developed^12^ a CNS‐penetrant small‐molecule drug candidate, MW01‐6‐189WH, hereafter called MW189. MW189 was developed as a selective suppressor of injury‐ and disease‐induced glial proinflammatory cytokine overproduction associated with destructive glial inflammation/synaptic dysfunction cycles, and their long‐term neurotoxic effects. A discovery approach based on the classic functional screening paradigm^13^, ^14^, ^15^, ^16^ was used to generate hits for medicinal chemistry optimization. The platform's early go–no go decision tree called for novel compounds possessing chemical and pharmacological properties to qualify for later development. Compounds were prioritized for the ability to restore dysregulated neuroinflammatory pathways back toward homeostasis without suppressing basal physiological cytokine levels at efficacious doses. For CNS discovery, attention was also paid at the front end on risk reduction features such as stability, blood‐brain barrier penetrance, avoidance of cytochrome P450 liabilities, and potential for safety.^17^, ^18^ Several candidates emerged from the platform, including MW189 and a close structural analogue MW01‐2‐151SRM (MW151).^12^ A summary of MW189 properties is shown in Supplemental Table S1.

MW189 is efficacious in vivo in animal models of acute brain injury, in which upregulation of proinflammatory molecules is implicated in disease progression, including TBI and ICH.^13^ For example, MW189 administered postinjury in mouse models of TBI and ICH reduced injury‐induced microglial activation and neuronal degeneration, as well as vestibular and cognitive deficits and edema.^13^ MW189 was effective at low doses (1 mg/kg), even when the initial dose was delayed until 6 hours after injury, demonstrating its feasibility to be used clinically during relevant injury‐to‐trauma center treatment time windows. By attenuating the inflammatory responses of overstimulated glia, MW189 may limit the pathological progression and neurocognitive dysfunction that complicate a variety of CNS disturbances. In addition, MW189's selective attenuation of upregulated biosynthetic processes such as proinflammatory cytokine production allows the potential of an extended pharmacodynamic (PD) effect compared to the time course of detectable drug levels. For example, acute administration of MW189 to injured mice during a limited time coincident with increasing cytokine production (hours to days postinjury) leads to improvements in neurologic end points evidenced weeks later, long after drug levels are undetectable.^19^

Based on these results, MW189 was taken forward into further preclinical safety and toxicology studies. Because critical care and severely injured emergency patients are not generally treated orally, an intravenous formulation of MW189 was developed based on the proposed route of administration to be used in clinical studies. Preclinical safety pharmacology and toxicology studies done with MW189 showed no treatment‐related adverse effects (Supplemental Table S1). Specifically, the safety pharmacology studies showed no adverse effects on vital organ systems (rat CNS, dog cardiovascular/respiratory). In a standard battery of tests for genotoxicity, MW189 was found to be neither mutagenic nor clastogenic. In repeated dose toxicology studies of twice‐daily intravenous dosing in rats and dogs for 14 days or once‐daily oral dosing in rats and dogs for 28 days, MW189 was well tolerated with no obvious adverse effect levels at the highest doses tested. Importantly, considering the anticipated pharmacological effect of MW189, no alteration in standard immunological parameters (hematology/blood cell counts, organ weights, macro‐ and microscopic evaluation of lymphoid and related tissues, etc.) was observed with repeat dosing.

Based on the pharmacologic properties, preclinical efficacy, and safety and toxicology profile of MW189, the decision was made to further develop MW189 for treatment of human acute brain injuries. We report here first‐in‐human studies of MW189 in healthy adult volunteers. This represents the first evaluation of the safety, tolerability, and pharmacokinetics (PK) in humans in both single‐ascending‐dose (SAD) and multiple‐ascending‐dose (MAD) phase 1 studies. We also report the results of a pilot phase 1 study to evaluate the PD effects of MW189 on endotoxin‐induced changes in blood cytokine levels in healthy volunteers. The results suggest engagement of pharmacological mechanism in humans, and provide support for further studies of MW189 for acute brain injury indications.

## Methods

All protocols were approved by the US Food and Drug Administration under an investigational new drug application and by the responsible institutional review board (IRB). The responsible IRB for the SAD was Aspire IRB (La Mesa, California), for the MAD study it was Duke University Health System IRB, for the endotoxin study it was Copernicus IRB (Research Triangle Park, North Carolina) followed by an administrative review by Duke University Health System IRB. The clinical research organization for the SAD study was the Parexel International Early Phase Clinical Unit, Baltimore, Maryland, and for the MAD and endotoxin studies it was the Duke Early Phase Clinical Research Unit, Duke University, Durham, North Carolina. All subjects were informed of the nature and purpose of the study, and their written informed consent was obtained before any study‐related procedures were performed. Studies were conducted in accordance with the principles set forth in the Declaration of Helsinki, the International Conference on Harmonization Tripartite Guidance on Good Clinical Practice, and the requirements of the Health Insurance Portability and Accountability Act of 1996, privacy regulations, and other applicable regulatory requirements including 21 Code of Federal Regulations 312.

### Name and Description of Investigational Product

**Figure 1:**
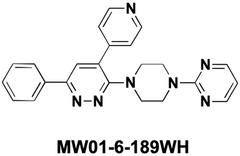
Chemical structure of MW189. 6‐Phenyl‐4‐(pyridin‐4‐yl)‐3‐(4‐(pyrimidin‐2‐yl)piperazin‐1‐yl)pyridazine (CAS #886208‐76‐0) has an empirical formula of C_23_H_21_N_7_ and a molecular weight of 395.47.

MW189 (Figure 1), 6‐phenyl‐4‐(pyridin‐4‐yl)‐3‐(4‐(pyrimidin‐2‐yl)piperazin‐1‐yl)pyridazine, has an empirical formula of C_23_H_21_N_7_ and a molecular weight of 395.47. The MW189 drug substance is a hydrochloride hydrate that is a light yellow to orange powder that is soluble in water. The drug product is a sterile concentrated solution in 0.9% sodium chloride (2.5 mg base/mL; pH, 2.4) that is diluted with saline prior to intravenous administration at pH > 3.0.

### Eligibility Criteria

#### Phase 1 SAD and MAD

Healthy male and female subjects aged 18 to 50 years with weight > 50 kg were eligible for the study. Volunteers were required to have adequate peripheral forearm vein access, not be pregnant or lactating, and agree to abstinence and acceptable contraceptive measures or have evidence of prior surgical sterilization, no prescription medication (except contraception) or over‐the‐counter medications or herbal/vitamin supplements (except acetaminophen ≤ 1 g/day and stable nonglucocorticoid treatment of seasonal allergies) in the 7 days prior to study entry, no use of nicotine‐containing products during the study, no current or recent (within 12 months) history of alcohol or drug abuse, no history of recent blood donation within 30 days of consenting or plasma donation within 60 days of consenting, and no previous participation in a clinical trial of an immunosuppressive drug or an investigational drug within 6 months of the study. Volunteers with significant medical or psychiatric illness by history, examination, or clinical laboratory testing that would influence study results or preclude informed consent and study compliance were excluded.

#### Phase 1 Endotoxin Study

Healthy male subjects aged 18 to 40 years with weight of 55‐95 kg were eligible for the study. Subjects had similar inclusion and exclusion criteria as above, as well as a requirement for a normal stable baseline body temperature on study day 1.

### Clinical Study Designs

#### Phase 1 SAD and MAD

This was a phase 1 randomized, double‐blind, placebo‐controlled single‐site study to determine the safety, tolerability, and PK of MW189 administered intravenously in a SAD and MAD design. In the first‐in‐human phase 1a SAD study, subjects underwent a screening visit to determine eligibility within 28 days of dosing. If eligible, subjects were admitted to the clinical research unit on the day prior to dosing (day ‐1). Dosing took place on day 1, and the subjects underwent safety and PK assessments for 48 hours postdose. Subjects were discharged on the morning of day 3 and returned for a safety follow‐up visit on day 8.

Four escalating doses of MW189 (0.025, 0.05, 0.10, and 0.25 mg/kg administered by intravenous infusion over 15‐20 minutes) were evaluated sequentially, starting with the lowest dose and escalating progressively to the highest dose. Eight subjects were dosed in each cohort, randomized to 6 MW189:2 placebo control. Matched placebo is 0.9% sodium chloride (saline). Dosing within each cohort was staggered, with a sentinel pair (1 MW189:1 placebo) dosing at least 1 day before the remaining cohort subjects (5 MW189:1 placebo). Dosing of the remaining cohort subjects was contingent on an acceptable safety review of the sentinel pair's day 1 safety data and 24‐hour postdose laboratory safety tests. Escalation from one cohort to the next was contingent on joint approval by the Investigator and sponsor following a comprehensive blinded safety review of the residential phase data.

In the MAD study, subjects were screened as above, admitted to the clinical research unit on day ‐1, and remained in the phase 1 unit until discharge on day 8. Follow‐up was done through a clinic visit at 2 weeks and a phone call at 4 weeks. Eight subjects were randomized to MW189 or saline placebo control (6 MW189:2 placebo). Subjects received 2 doses per day for 5 consecutive days, administered by intravenous infusion over 15‐20 minutes and given 12 hours apart. Four dose cohorts were evaluated: 0.075, 0.15, 0.25, and 0.30 mg/kg intravenously twice daily (or matched placebo).

#### Phase 1 Endotoxin Study

This was a phase 1 double‐blind, randomized, placebo‐controlled study to evaluate the effects of a single intravenous dose of MW189 on endotoxin‐induced changes in plasma cytokine levels in healthy male volunteers. Eighteen adult subjects were randomly assigned to 1 of 2 treatment groups (MW189 or placebo), in a 1:1 ratio. All 18 completed the study. All subjects who received any study treatment were included in the safety analysis, and all subjects who received the intended dose of lipopolysaccharide (n = 16) were included in the cytokine analysis.

On day ‐1, each subject underwent a thorough safety review to confirm that they were healthy, stable, and eligible for the study. Subjects provided a safety laboratory sample, and a hydration program was initiated to ensure adequate hydration on day 1. On day 1, each subject received a single intravenous dose of study drug (0.25 mg/kg MW189 or matched placebo) administered as a 20‐minute infusion followed immediately by a single‐bolus intravenous injection of low‐dose endotoxin (*Escherichia coli* lipopolysaccharide [LPS]) at 2 ng/kg in the contralateral arm.

Careful monitoring of standard safety parameters after LPS administration occurred, including evaluation of adverse events (AEs), vital signs, electrocardiograms (ECGs), and body temperature. Blood samples for cytokine measurements were collected over a 12‐hour period following the LPS challenge. Subjects were discharged on the morning of day 2, after completion of the 24‐hour assessments and with the Investigator's approval and returned on day 6 for a follow‐up visit.

### Safety Evaluations and Analyses

Safety and tolerability were assessed during and following dosing by clinical staff and physician observation and spontaneous reporting of symptoms by subjects. Other safety and tolerability end points included reported AEs, changes in vital signs, physical and neurological examination results, 12‐lead ECGs, and clinical laboratory tests including hematology, chemistry, and urinalysis. A safety monitoring committee evaluated the safety and tolerability in the dose‐escalation cohorts. Safety data for each dosing cohort were reviewed prior to initiation of each subsequent cohort. Doses were escalated in successive cohorts unless 1 subject experienced a serious adverse event (except if clearly unrelated to the study product) or 2 participants reported the same adverse event of moderate intensity that was considered at least probably related to the study drug.

All subjects who received any study treatment were included in the safety analysis grouped by treatment received. The statistical analysis of safety data is descriptive in nature; no inferential hypothesis testing was performed on the safety variables. For all safety analyses, baseline was defined as the last evaluation before dosing. For continuous variables, summaries include sample size, mean, standard deviation, minimum, and maximum. For categorical variables, the summaries include frequencies and corresponding percentages. Repeat or unscheduled results were not included in the summaries, but are listed. Data from subjects who received placebo were pooled for data presentations. Statistical analyses of these data were performed using version 9.1 (SAD) or version 9.4 (MAD) of SAS (Cary, North Carolina).

### Drug Concentration (Pharmacokinetic) Measurements

#### Blood Sampling and Plasma Preparation

Blood samples were obtained from each subject in lithium heparin tubes for the determination of MW189 plasma concentrations. For the SAD cohorts, blood samples were collected prior to dosing (0 hours) and at the following postdose times: 7 minutes, end of infusion (15 or 20 minutes), 0.5, 1, 2, 4, 8, 12, 36, and 48 hours postdose. For the MAD cohorts, blood samples were collected at the following times, anchored to the start of each infusion:

- *Day 1 morning dose*: predose, 20 minutes (at the end of infusion; sample collected prior to completion of the infusion), 1, 2, 4, 7.5, and 12 hours (immediately before the day 1 second dose) postdose.
- *Day 1 second dose*: 20 minutes (at the end of infusion; sample collected prior to completion of the infusion) 1, 2, 4, and 7.5 hours postdose.
- *Day 2 morning dose*: predose (approximately 24 hours after day 1 morning dose).
- *Day 3 morning dose*: predose (approximately 48 hours after day 1 morning dose).
- *Day 4 morning dose*: predose (approximately 72 hours after day 1 morning dose).
- *Day 5 morning dose*: predose (approximately 96 hours after day 1 morning dose), 20 minutes (at the end of infusion; sample collected prior to completion of the infusion), 1, 2, 4, 7.5, and 12 hours (immediately before the day 5 second dose) postdose.
- *Day 5 second dose*: 20 minutes (at the end of infusion; sample collected prior to completion of the infusion), 1, 2, 4, and 7.5 hours postdose.
- *Day 6*: approximately 16 hours after day 5 second dose, approximately 24 hours after day 5 second dose.
- *Day 7*: approximately 48 hours after day 5 second dose.

Following collection, blood samples were immediately placed on ice, plasma was separated by centrifugation, transferred to polypropylene specimen containers, and frozen at ‐70°C until shipped to the bioanalytical laboratory (Biovail Contract Research/Lambda Therapeutic Research, Toronto, Ontario, Canada, for the SAD study; MPI Research, Mattawan Michigan, for the MAD study).

#### Bioanalytical Methods

Plasma concentrations of MW189 were determined with a validated bioanalytical assay employing liquid chromatography‐tandem mass spectrometry (LC‐MS/MS). The assay was validated over the concentration range of 0.08 to 20.0 ng/mL (±10%). An internal standard was used to normalize samples in terms of sample handling and LC‐MS/MS method precision. For the SAD, the internal standard was MW151,^12^ a close structural analogue of MW189; for the MAD, the internal standard was either MW151 or a deuterated form of MW151 (MW151‐D_4_). For both the SAD and MAD, transitions of the respective [M‐H]^+^ ions were used to monitor MW189 (*m/z* 396 → 275), MW151 (*m/z* 333→ 212), and MW151‐D_4_ (*m/z* 337→ 214). Long‐term stability studies showed that MW189 was stable in human plasma for at least 380 days when stored at ‐70°C and up to 231 days when stored at −25°C. All PK samples were analyzed within this established storage stability period.

For the SAD, extraction of MW189 and the internal standard from human plasma was done by protein precipitation in methanol: 0.1% formic acid (50:50, v/v). The analytes were separated by high‐pressure liquid chromatography using reversed‐phase chromatography on a Thermo Hypersil Gold column (100 × 2.1 mm, 5.0 µm) at room temperature. The mobile phase was methanol: 0.005 ammonium formate with 0.1% formic acid (65:35, v/v); run time was ∼4.5 minutes. Detection was done using a TSQ Quantum tandem mass spectrometer with electrospray ionization (ESI) source operating in the positive ion mode. Intra‐assay precision range (%CV) was 2.4%‐6.9%, and interassay %CV was 2.7%‐5.8%.

For the MAD, extraction of MW189 and the internal standard from human plasma was done in methanol, then 100 µL of the supernatant was mixed with 100 µL of water/methanol/formic acid (50/50/0.1, v/v/v), and injected onto the LC‐MS/MS system for analysis. The LC system used a Phenomenex Kinetex C18 column (2.1 × 50 mm, 2.6 µm) with an isocratic flow consisting of water/1M ammonium formate/formic acid (100/0.5/0.1, v/v/v) and methanol at a flow rate of 0.4 mL/min. Analytes were detected using a SCIEX API 5000 triple quadrupole LC‐MS/MS system equipped with an ESI (TurboIonSpray) ionization source operated in the positive ion mode. Intraassay %CV was 2.0% to 9.8%, and interassay %CV was 2.6% to 5.4%.

#### Pharmacokinetic Analyses

Individual plasma concentration‐versus‐time profiles of MW189 for the SAD and MAD studies were used to generate PK parameters using noncompartmental analysis. The noncompartmental PK analysis was performed in Phoenix WinNonlin (version 5.2 [SAD] or version 6.3 [MAD]; Pharsight Corporation, Mountain View, California). An assessment of steady state was performed by comparing trough concentrations (C_MIN_) following doses 1, 2, 3, 5, 7, and 9 using the Helmert transformation methodology,^20^ a method for determining time to steady state. In this method, the mean concentration at the first time is compared with the pooled mean over all remaining times, then the mean at the second time is compared with the pooled mean over all remaining times, and then testing continues in this way until the contrast is not statistically significant. The first dose in the first nonsignificant contrast was considered the time at which steady state was attained. Within‐participant correlation was modeled using a linear mixed‐effects model with participant as a random effect. The model assumed a monotonic nondecreasing function with balanced data. Any concentration below the quantification limit was assigned a value of zero for the PK calculations.

For both the SAD and MAD cohorts, the peak drug concentration (C_max_) and time of peak concentration (T_max_) were calculated using the observed data for MW189. For the SAD cohort, AUC from time 0 to time of last nonzero concentration (AUC_0‐t_), AUC from 0 extrapolated to infinity (AUC_0‐inf_), and elimination half‐life (t_1/2_) were calculated. For the MAD cohort, AUC from 0 to 12 hours (AUC_tau_) was assessed for MW189. AUC_0‐t_ and AUC_tau_ were calculated using the linear trapezoidal method. Trough concentration (C_MIN_), defined as the concentration 12 hours after the last dose and before start of the next dose was calculated using the observed data for MW189 after doses 1 and 2 on day 1, dose 1 on days 2, 3, and 4, and dose 1 on day 5. Using data from the last dosing on day 5, the following additional PK parameters were estimated: terminal elimination rate constant (λz), elimination half‐life (t_1/2_), clearance (CL), and volume of distribution during the terminal phase (V_z_). λz was determined as the slope of a log‐linear least squares of at least 3 concentration‐time points judged after the last dose by visual inspection to be in the apparent terminal elimination phase. Half‐life was calculated as t_1/2_ = ln_2_/ λz. Clearance at steady state (CL_SS_) was estimated using CL_SS_ = dose/AUC_tau_. V_z_ was estimated using V_z_ = dose/(λ_z_ × AUC_0‐inf_). Area under the concentration‐time curve from time of last dosing to infinity (AUC_0‐inf_) was estimated using AUC_0‐inf_ = AUC_LAST_ + C_LAST_/λ_z_, where C_LAST_ was the last measurable plasma concentration after the last dose and AUC_LAST_ was the AUC from the time of last dosing to the time of the last measurable sample. AUC_LAST_ was calculated using the linear trapezoidal method, and at least 3 times after the last dose with measurable plasma concentrations were required for the calculation of AUC_LAST_. Values for λz and other λz‐related parameters (eg, AUC_0‐inf_, t_1/2_, CL, V_Z_) were not reported for concentration profiles that did not exhibit a terminal elimination phase in the concentration‐versus‐time profile. Nominal sampling time was used in PK calculations.

### Pharmacodynamic Cytokine Analyses

Blood samples were collected in potassium‐ethylenediaminetetraacetic acid tubes predose on day 1 and at 30 minutes and 1, 1.5, 2, 3, 4, 6, 8, and 12 hours post‐LPS administration. Plasma was separated by centrifugation, and frozen at ‐70°C until shipped to the cytokine testing laboratory for analysis. Plasma levels of the cytokines interleukin (IL)‐1ra, IL‐6, IL‐8, IL‐10, tumor necrosis factor‐α (TNF‐α), and chemokine C‐C motif ligand 2 (CCL2) were measured by a CLIA‐certified biomarker testing service (Aushon BioSystems, Inc., Billerica, Massachusetts), using their SearchLight Custom Array Technology.^21^ The SearchLight microplate‐based array is a multiplex sandwich enzyme‐linked immunosorbent assay in which capture antibodies are spotted in single wells and the secondary detection antibody is conjugated with a chemiluminescent label. Three custom arrays were used ([1] TNF‐α/IL‐6/IL‐8/IL‐10, [2] CCL2, [3] IL‐1ra), grouped according to their expected plasma concentrations. Analyte concentrations were quantified by comparison with the corresponding standard curves that had previously been qualified over the linear concentration ranges for each analyte.

For each cytokine, the differences in mean cytokine levels were assessed for treatment by time (treatment × time) effects using the generalized linear model procedure (analysis of variance) in SAS on ln‐transformed data. If the treatment × time effect was found to be statistically significant, then treatment levels were compared for each time separately using post hoc analysis (SLICE option from the least‐squares means [LSMEANS] statement). The SLICE option provides a test of the equality of means for each level of each effect in an interaction. The analysis tested for a treatment‐by‐time effect sliced by time to determine at which times the significant differences occurred. *P* values were derived from type III sums of squares. All inferential statistical analyses were performed at the 0.05 level of significance.

## Results

### Disposition of Subjects

In the SAD study, 32 subjects were randomized (8 placebo and 24 MW189). There were no discontinuations, and all subjects completed the study. In the MAD study, a total of 35 subjects were randomized (9 placebo and 26 MW189), and 28 completed the study. Seven subjects prematurely discontinued: 3 placebo (33.3%; 1 each for AE, lost to follow‐up, and withdrawal by subject) and 4 MW189 (15.4%; 2 each for AE and withdrawal by subject), all of whom were assigned to receive 0.30 mg/kg MW189. Of the 4 0.30 mg/kg MW189 subjects, 2 were replaced to yield a total of 8 subjects in the 0.30 mg/kg dose group. In the endotoxin study, 18 healthy adults were enrolled, and all completed the study.

### Demographics

Summary demographic data and baseline characteristics for subjects in the SAD and MAD studies are shown in Table 1. Subjects were relatively well matched for weight, height, and body mass index across treatment and dose groups. The overall SAD study population (Table 1a) was composed of 27 male and 5 female subjects; among MW189 subjects, 19 were male and 5 were female, and the placebo group was 8 male and 0 female subjects. The mean age of all subjects treated with MW189 was 31.2 years, and the mean of subjects treated with placebo was 37.8 years. Reflecting the demographics of the clinical site, most subjects in each treatment group were black/African American (75.0% placebo, 79.2% MW189), and all but 2 subjects were non‐Hispanic or Latino. The MAD study population (Table 1b) showed similar demographics, to the study population composed of 19 male and 15 female subjects; among MW189 subjects, 16 were male and 10 were female, and the placebo group was 3 male and 5 female subjects. The mean age of all subjects treated with MW189 was 32.1 years, and the mean of subjects treated with placebo was 37.4 years. Most subjects in each treatment group were black/African American (62.5% placebo, 57.7% MW189), and all but 3 subjects were non‐Hispanic or Latino.

**Table 1:** Demographic Summary of SAD and MAD Studies

		MW189 Treatment Group				
1a. SAD Study	Placebo	0.025 mg/kg	0.05 mg/kg	0.10 mg/kg	0.25 mg/kg	All Active
Parameter	n = 8	n = 6	n = 6	c = 6	n = 6	n = 24
Continuous, mean (SD)						
Age (years)	37.8 (7.4)	28.2 (5.2)	30.2 (9.1)	28.5 (7.4)	38.0 (5.8)	31.2 (7.7)
Height (cm)	175.9 (5.0)	172.7 (3.1)	175.8 (7.1)	178.8 (7.0)	168.5 (9.0)	174.0 (7.6)
Weight (kg)	76.5 (10.8)	81.4 (10.8)	73.8 (11.3)	73.6 (6.2)	86.4 (9.6)	78.8 (10.6)
BMI (kg/m^2^)	24.7 (2.9)	27.3 (3.2)	23.9 (3.3)	23.2 (2.9)	30.4 (2.8)	26.2 (4.1)
Categorical, n (%)						
Race						
Black/African American	6 (75.0)	5 (83.3)	4 (66.7)	6 (100.0)	4 (66.7)	19 (79.2)
White	2 (25.0)	0 (0.0)	2 (33.3)	0 (0.0)	2 (33.3)	4 (16.7)
American Indian/Alaska Native	0 (0.0)	1 (16.7)	0 (0.0)	0 (0.0)	0 (0.0)	1 (4.2)
Ethnicity						
Hispanic	0 (0.0)	0 (0.0)	0 (0.0)	0 (0.0)	2 (33.3)	2 (8.3)
Non‐Hispanic or Latino	8 (100.0)	6 (100.0)	6 (100.0)	6 (100.0)	4 (66.7)	22 (91.7)
Sex						
Female	0 (0.0)	1 (16.7)	2 (33.3)	0 (0.0)	2 (33.3)	5 (20.8)
Male	8 (100.0)	5 (83.3)	4 (66.7)	6 (100.0)	4 (66.7)	19 (79.2)

		MW189 Treatment Group				
1b. MAD Study	Placebo	0.075 mg/kg	0.15 mg/kg	0.25 mg/kg	0.30 mg/kg	All Active
Parameter	n = 8	n = 6	n = 6	n = 6	n = 8	n = 26
Continuous, mean (SD)						
Age (years)	37.4 (6.7)	32.4 (5.9)	31.6 (6.2)	34.5 (7.4)	30.5 (10.9)	32.1 (7.8)
Height (cm)	165.8 (6.7)	171.6 (8.7)	164.0 (11.1)	170.9 (10.5)	173.1 (8.4)	170.1 (9.9)
Weight (kg)	74.5 (6.1)	70.5 (9.1)	75.2 (6.2)	85.5 (20.5)	86.4 (12.7)	79.9 (14.2)
BMI (kg/m^2^)	27.1 (1.9)	24.1 (3.8)	28.1 (2.7)	29.1 (5.6)	28.8 (3.8)	27.6 (4.3)
Categorical, n (%)						
Race^a^						
Black/African American	5 (62.5)	5 (83.3)	2 (33.3)	3 (50.0)	5 (62.5)	15 (57.7)
White	4 (50.0)	1 (16.7)	4 (66.7)	2 (33.3)	3 (37.5)	10 (38.5)
American Indian/Alaska Native	1 (12.5)	0 (0.0)	2 (33.3)	0 (0.0)	0 (0.0)	2 (7.7)
Asian	0 (0.0)	1 (16.7)	1 (16.7)	1 (16.7)	0 (0.0)	3 (11.5)
Ethnicity						
Hispanic or Latino	1 (12.5)	0 (0.0)	1 (16.7)	0 (0.0)	1 (12.5)	2 (7.7)
Non‐Hispanic or Latino	7 (87.5)	6 (100.0)	5 (83.3)	6 (100.0)	7 (87.5)	24 (92.3)
Sex						
Female	5 (62.5)	1 (16.7)	4 (66.7)	1 (16.7)	4 (50)	10 (38.5)
Male	3 (37.5)	5 (83.3)	2 (33.3)	5 (83.3)	4 (50)	16 (61.5)

n, number of subjects in cohort; SD, standard deviation; %, percentage of subjects in cohort; BMI, body mass index (defined as weight in kilograms divided by height in meters squared).

a Multiple races were reported in some subjects, so the sums of the race numbers do not always match the group numbers.

### Safety and Tolerability

#### SAD

MW189 was well tolerated at single doses of 0.025, 0.05, 0.10, and 0.25 mg/kg in healthy adult male and female subjects. There were no treatment‐emergent adverse events (TEAEs) leading to withdrawal, no deaths, and no serious adverse events (SAEs) during the study. Overall, 7 TEAEs were reported by 5 subjects (2 placebo, 3 MW189). The incidence of TEAEs was higher in the placebo group (25% of subjects reporting) than in the MW189 group (12.5% of subjects reporting). All the reported AEs were mild in severity and resolved completely without medical intervention. Two subjects reported 3 TEAEs, which were considered possibly related to the study drug (1 placebo subject reported headache, 1 MW189 subject reported headache and nausea). There was no pattern of increasing incidence of TEAEs with increasing MW189 dose. No laboratory, vital sign, ECG, or injection‐site reaction AEs were reported during the study, and no treatment‐related or dose‐related trends were observed.

#### MAD

Overall, multiple ascending doses of MW189 were safe and well tolerated, with no deaths or SAEs reported. The overall incidence of TEAEs was higher in the all MW189 treatment group (84.6%) than in the placebo treatment group (62.5%). However, these reported TEAEs were all mild to moderate in intensity and generally transient in nature. There was no pattern of increasing incidence of TEAEs with increasing MW189 dose. The incidence of TEAEs related to the study drug was also higher in the all MW189 treatment groups (69.2%) than in the placebo treatment group (37.5%). This imbalance was driven by an increase of mild to moderate infusion‐related AEs (pain in extremity, infusion‐site pain, phlebitis) in the higher MW189 dose groups. With regard to infusion‐associated AEs, the 0.25 mg/kg dose appeared better tolerated than the 0.30 mg/kg dose. One subject in the placebo treatment group and 3 subjects in the 0.30 mg/kg MW189 dose group experienced a TEAE that led to study drug discontinuation. Only 1 subject (in the 0.30 mg/kg MW189 dose group) had a discontinuation event considered related to the study drug (pain in extremity), although another subject in the 0.30 mg/kg MW189 dose group had TEAEs considered related to the study drug that may have contributed to a decision to withdraw from the study. Postdose changes in laboratory, vital sign, and ECG values were small, transient, and not considered clinically significant. No treatment or dose‐related trends were observed. None of the subjects had a postdose clinically significant finding on physical or neurological examination.

#### Endotoxin Study

All the subjects received LPS and therefore exhibited typical flu‐like symptoms, but the intensity and duration varied. The low‐dose endotoxin exposure was well tolerated, with the incidence rates of clinical signs (fever, vital signs) and symptoms (chills, headache, nausea, myalgia) of inflammation being similar between placebo and the MW189 treatment groups. There were no SAEs, severe AEs, or unexpected TEAEs during the study. No clinically relevant changes in laboratory parameters, vital signs, or ECG findings were observed beyond those anticipated with LPS. The incidence rates of AEs were similar in the placebo and MW189 treatment groups.

### Pharmacokinetics

#### SAD

**Figure 2:**
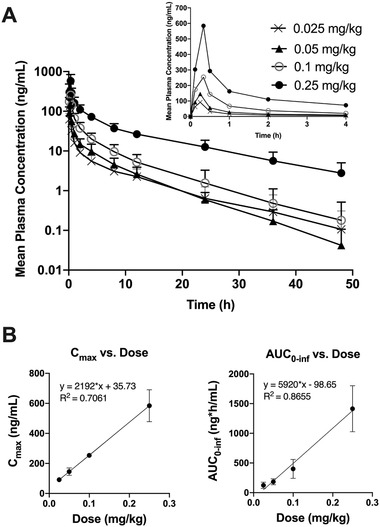
MW189 plasma concentration‐time profiles and dose‐proportional increases in exposure following single ascending doses of MW189. Four escalating doses (0.025, 0.05, 0.10, 0.25 mg/kg) of MW189 were administered as a single intravenous infusion. (A) The mean ± SD concentrations (ng/mL) of MW189 versus time after the dosing (hours) are plotted on a log‐linear scale. The inset graph shows an expansion of the data for the first 4 hours after dosing, plotted on a linear scale. (B) The mean ± SEM of C_max_ (maximum observed concentration) and AUC_0‐inf_ (area under the concentration‐time curve from time zero to infinity [extrapolated]) parameters are plotted versus increasing single doses of MW189. Linear regression analyses are consistent with dose proportionality.

Plasma concentration‐time profiles (Figure 2A) and plasma exposure (Table 2) increased following intravenous administration of increasing single doses of 0.025, 0.05, 0.10, and 0.25 mg/kg. The time to maximum plasma concentration (T_max_) was 15‐20 minutes for all dose levels tested, corresponding to the plasma sampling time taken at the end of the MW189 infusion. The mean C_max_ and AUC values observed across all dose groups suggested proportional increases in exposure with increasing dose (Figure 2B). The T_1/2_ for MW189 following intravenous administration (∼6 hours) was similar to that of dogs (∼4‐5 hours) and appeared slightly higher (∼10 hours) for the highest dose group. The CL and V_z_ were comparable across doses. Thus, after a single intravenous dose of 0.025 to 0.25 mg/kg, MW189 exhibited approximately linear kinetics and dose‐proportional PK, suggesting first‐order clearance mechanisms in the dose range studied.

**Table 2:** Plasma PK Parameter Estimates for MW189 in the SAD Study Stratified by Dose

	Dose of MW189			
Parameter (Units)	0.025 mg/kg	0.05 mg/kg	0.10 mg/kg	0.25 mg/kg
T_max_ (h)	0.25 ± 0.0	0.27 ± 0.05	0.33 ± 0.0	0.33 ± 0.0
C_max_ (ng/mL)	91.4 ± 20.8	145 ± 59.6	254 ± 35.2	584 ± 261
AUC_0‐t_ (ng·h/mL)	121 ± 49.8	182 ± 48.8	397 ± 156	1360 ± 355
AUC_0‐inf_ (ng·h/mL)	124 ± 52.4	183 ± 49.4	400 ± 160	1410 ± 385
T_1/2_ (h)	6.90 ± 2.79	6.12 ± 1.57	6.24 ± 1.86	10.37 ± 3.47
Cl (L/h/kg)	0.23 ± 0.09	0.29 ± 0.08	0.27 ± 0.07	0.19 ± 0.07
V_z_ (L/kg)	2.05 ± 0.35	2.58 ± 0.91	2.33 ± 0.41	2.68 ± 0.66
V_ss_ (L/kg)	1.26 ± 0.25	1.31 ± 0.44	1.18 ± 0.24	1.85 ± 0.57

T_max_, time to maximum plasma concentration; C_max_, maximum observed concentration; AUC_0‐t_, area under the concentration‐time curve from time zero to time of last nonzero concentration; AUC_0‐inf_, area under the concentration‐time curve from time zero to infinity (extrapolated); T_1/2_, elimination half‐life; Cl, total body clearance; V_z_, volume of distribution; V_ss_, estimated volume of distribution at steady state.

Data are shown as mean ± standard deviation.

#### MAD

**Figure 3:**
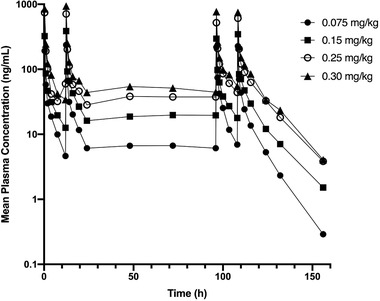
MW189 plasma concentration‐time profiles following multiple ascending doses of MW189. Four escalating doses (0.075, 0.15, 0.25, and 0.30 mg/kg) of MW189 were administered by intravenous infusion every 12 hours over 5 days. The mean concentrations (ng/mL) of MW189 versus time after the dosing (hours) are plotted on a log‐linear scale.

Plasma concentration‐time profiles (Figure 3) and associated PK parameters (Table 3) are shown for the 4 MAD dose cohorts (0.075, 0.15, 0.25, and 0.30 mg/kg). MW189 displayed approximately linear kinetics with dose‐proportional increases in C_max_ and AUC_tau_ in all dosing groups. T_1/2_ was comparable across the doses, ranging from approximately 3.8 to 9.1 hours. Steady state was achieved following the second dose in the 0.075 and 0.15 mg/kg cohorts based on nonsignificant difference in C_min_ values for subsequent doses (Supplemental Table S2). Steady state was achieved after the third dose in the 0.25 and 0.30 mg/kg cohorts.

**Table 3:** Plasma PK Parameter Estimates for MW189 in the MAD Study Stratified by Dose

	Dose of MW189							
	0.075 mg/kg		0.15 mg/kg		0.25 mg/kg		0.30 mg/kg	
	n = 6	n = 6	n = 6	n = 6	n = 6	n = 6	n = 8	n = 4
Parameter (Units)	Dose 1	Dose 10	Dose 1	Dose 10	Dose 1	Dose 10	Dose 1	Dose 10
C_max_ (ng/mL)	192 ± 89	227 ± 80.5	323 ± 89.4	347 ± 86.8	743 ± 210	609 ± 180	851 ± 188	750 ± 235
AUC_tau_ (ng·h/mL)	275 ± 63.9	362 ± 123	474 ± 132	639 ± 220	912 ± 278	1225 ± 474	1222 ± 185	1525 ± 458
T_1/2_ (h)	3.8 ± 1.7	8.5 ± 3.4	6.6 ± 3.4	8.5 ± 3.7	7.4 ± 2.9	8.8 ± 2.8	6.4 ± 2.3	9.1 ± 2.8
Cl_ss_ (L/h/kg)	0.286 ± 0069	0.229 ± 0.082	0.335 ± 0.081	0.256 ± 0.073	0.299 ± 0.104	0.236 ± 0.103	0.251 ± 0.041	0.210 ± 0.061
V_z_ (L/kg)	1.48 ± 0.44	2.92 ± 1.91	2.97 ± 1.23	2.84 ± 0.52	2.96 ± 0.98	2.72 ± 0.63	2.34 ± 0.94	2.72 ± 0.63

C_max_, maximum observed concentration; AUC_tau_, area under the concentration‐time curve from time zero to 12 hours after dose 1; T_1/2_, elimination half‐life; Cl_ss_, steady state clearance; V_z_, volume of distribution; n, number of participants.

Data are shown as mean ± standard deviation.

### Pharmacodynamic (Cytokine) Analyses

The pharmacological mechanism of action of MW189 in preclinical studies is to attenuate stressor‐induced upregulation of proinflammatory cytokines. It was of interest, therefore, to explore whether MW189 could show this PD effect in humans. However, using cytokines as biomarkers of a PD effect in healthy young adult subjects is not feasible, as cytokine levels are constitutively low in the absence of an inflammatory trigger, and these basal physiological levels of cytokines are not suppressed by MW189. For this reason and because of the reproducibility and predictability of the LPS endotoxemia model to induce a transient acute inflammatory response, administration of low levels of endotoxin to healthy volunteers is accepted as a safe model to study mechanisms and treatments for systemic inflammation.^22^, ^23^ Although the role of cytokines in neuroinflammation after acute brain injury differs both quantitatively and qualitatively from the systemic inflammatory response induced by LPS, this pilot experiment was undertaken as an initial step to explore the anti‐inflammatory potential of MW189 in humans.

**Figure 4:**
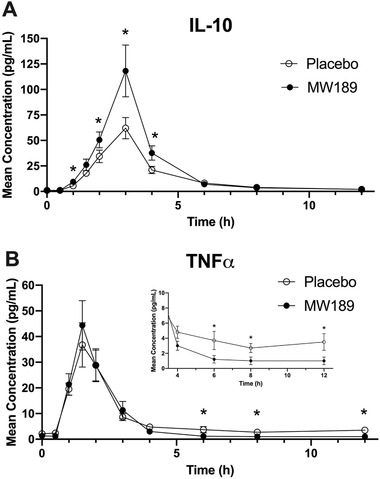
Plasma cytokine levels following endotoxin challenge and MW189 administration. Healthy adult male volunteers were administered a single intravenous dose of MW189 (0.25 mg/kg) or matched placebo, followed immediately by a single intravenous injection of low‐dose endotoxin (LPS, 2 ng/kg) to induce increases in plasma cytokine levels. Blood was collected over a 12‐hour period after the LPS challenge, and plasma cytokines were measured by ELISA (8 MW189, 8 placebo). Mean ± SEM plasma levels (pg/mL) are shown for (A) IL‐10 and (B) TNF‐α. Note that MW189 treatment increases the anti‐inflammatory cytokine IL‐10 level and decreases the proinflammatory cytokine TNF‐α level compared with placebo treatment. The inset graph in (B) shows an expansion of the data for the last 4 times (4, 6, 8, and 12 hours). *Statistically significant difference between MW189 and placebo groups.

Of the 18 subjects in the study, 2 subjects (1 placebo and 1 MW189) received only partial LPS doses because of a mg/kg calculation error; therefore, only 16 subjects were included in the cytokine analyses. The mean cytokine level‐versus‐time profiles of the 6 cytokines measured in plasma over the first 12 hours after LPS administration (IL‐1ra, IL‐6, IL‐8, IL‐10, TNF‐α, CCL2) were similar between MW189‐ and placebo‐treated subjects, showing the anticipated LPS‐induced acute cytokine surge in the first few hours and then returning back toward baseline by 12 hours. Four of the cytokines (IL‐1ra, IL‐6, IL‐8, CCL2) showed no significant differences between MW189‐ and placebo‐treated subjects. However, there were statistically significant differences in the mean plasma levels of IL‐10 and TNF‐α in MW189‐treated versus placebo‐treated subjects (Supplemental Table S3). Specifically, the MW189 group showed significantly higher levels of the anti‐inflammatory cytokine IL‐10 compared with the placebo group; these differences between treatments occurred around the time of maximum effect, from 1 to 4 hours post‐LPS (Figure 4A). In addition, the MW189 group showed significantly lower levels of the proinflammatory cytokine TNF‐α from 6 to 12 hours post‐LPS compared with the placebo group (Figure 4B); these differences occurred on the 3 last sampling times, from 6 to 12 hours post‐LPS and were close to the baseline values.

## Discussion

This study reports the first clinical experience with MW189, a novel CNS‐penetrant small molecule that selectively suppresses injury‐ and disease‐induced glial proinflammatory cytokine overproduction.^12^ The phase 1a SAD and phase 1b MAD trials demonstrated that single and multiple intravenous doses of MW189 in healthy adult volunteers were well tolerated. MW189 treatment showed no clinical or laboratory safety signals of potential clinical concern. AEs were mostly mild, transient, and resolved without intervention. The primary TEAE‐related safety issue observed was mild to moderate drug‐related, infusion‐associated AEs (pain in extremity, infusion‐site pain, phlebitis), which occurred at a higher frequency in the highest MW189 dose group (0.30 mg/kg). These infusion‐related AEs were potentially because of the acidic composition of the study drug and the route of administration (intravenous infusions twice daily over 5 consecutive days). Because the infusion‐associated AEs were less tolerable at the highest dose studied (0.30 mg/kg), a future phase 2a study done with a similar intravenous administration will be done at a dose of 0.25 mg/kg. The method of intravenous drug delivery could also be modified, for example, slowing the flow or changing the route of delivery to a central line or a peripherally inserted central catheter, to potentially limit these events in future studies.

MW189 showed similar PK profiles after single or multiple administrations. When dose‐normalized, the C_max_ and AUC_tau_ values were comparable in both the SAD and MAD studies. The range of mean CL_ss_, V_z_, and T_1/2_ were also comparable between studies. The plasma concentration‐time profiles and associated PK parameters demonstrated dose‐proportional and approximately linear kinetics at all doses tested, suggesting first‐order clearance mechanisms across the entire dose range tested (0.025 to 0.30 mg/kg). The clearance and volume of distribution were not appreciably different between dose groups, and there was no significant accumulation of MW189. Steady state was achieved by dose 3 for all dosing cohorts. Overall, the PK parameters support a twice daily intravenous dosing regimen in future clinical studies.

Preclinical efficacy and PK studies of MW189 were performed in rats and permitted determination of PK parameters associated with an efficacious dose. Efficacy studies in a rat rheumatoid arthritis model of inflammation showed a significant reduction in inflammation and clinical disease with a minimum effective dose of 1 mg/kg (unpublished data). After dose‐normalizing the PK data to the minimum effective dose of 1 mg/kg, mean C_max_ and AUC_tau_ over the dosing interval associated with an effective dose ranged from 513 to 741 ng/mL and 496 to 694 ng·h/mL, respectively. These values were comparable to the mean C_max_ and AUC_tau_ following a dose of 0.25 and 0.15 mg/kg, respectively. Thus, doses of 0.15 to 0.25 mg/kg MW189 would achieve comparable exposures associated with efficacy in animal models, further supporting the use of 0.25 mg/kg dosing in future clinical studies.

To begin to explore engagement of the pharmacological mechanism in humans, a small pilot study was done to evaluate the effects of a single intravenous dose of MW189 on endotoxin‐induced changes in blood cytokine levels in healthy volunteers. This study was not powered for efficacy, but was designed as an initial screen in humans. The goal was to assess whether MW189 could induce any changes in plasma cytokine levels in volunteers administered low levels of endotoxin (LPS) to induce systemic inflammation. As anticipated with only 8 subjects per group, the LPS‐induced cytokine profile for most of the cytokines (IL‐1ra, IL‐6, IL‐8, CCL2) showed no significant differences between MW189‐ and placebo‐treated subjects. However, MW189 treatment led to significantly higher levels of the anti‐inflammatory cytokine IL‐10 and significantly lower levels of the proinflammatory cytokine TNF‐α compared with placebo treatment. These results have implications for future development of MW189. The results show that MW189 administered intravenously does not immunosuppress as judged by the ability of the subjects to respond to an LPS challenge by increases in plasma cytokine levels. In addition, the significant increase in IL‐10 and decrease in TNF‐α levels in MW189‐treated subjects compared with placebo may be a relevant observation in the context of the anti‐inflammatory mechanisms of the drug.

MW189 has several properties relevant to a pharmacological intervention for acute brain injuries. Its pharmacological mechanism of action is selective attenuation of injury‐induced upregulated biosynthesis of proinflammatory cytokines, and MW189 targets cytokine biosynthesis through pathways that are not dependent on p38α mitogen‐activated protein kinase (MAPK) activation. MW189 does not inhibit p38α MAPK or other standard kinases^18^, ^24^, ^25^ and does not show activity in assays that depend on p38α MAPK signaling.^26^ Secondary pharmacology screens of MW189 with 412 kinases and 55 G‐protein coupled receptors showed no binding (reference 18 and Table S1). MW189 is not a nonsteroidal anti‐inflammatory drug and is distinct from steroids or pansuppressors of diverse tissue responses. By targeting a biosynthetic process, MW189 has the potential of an extended PD effect compared with the time course of detectable drug levels. A key aspect of dosing is the therapeutic time window, which is driven by consideration of patient diagnosis and pathophysiology progression. This means that the pathological process targeted by the drug should be occurring during a time window that allows for presentation of the patient to the trauma center for therapeutic intervention. For acute brain injury, that intervention window is the acute cytokine surge that occurs in the first several hours to days after injury. Treatment with MW189 in preclinical injury models, even when initial drug administration was delayed until 6 hours postinjury, demonstrated the ability of this drug candidate to engage the pharmacological mechanism of action and bring about improvements in neurological outcomes.^13^ The pilot endotoxin study reported here is also consistent with the ability of MW189 to engage its pharmacological mechanism in humans and supports continued development of MW189 for acute brain injury.

## Conclusion

These first‐in‐human studies report the initial clinical experience with MW189, characterizing the overall safety and tolerability, PK, and initial PD of MW189 administered intravenously in healthy adult volunteers. Three major findings emerge from these studies. First, MW189 was safe and well tolerated over the dose ranges tested in both SAD and MAD studies, with no significant clinical or laboratory safety concerns. The primary TEAE‐related safety issue observed was infusion‐related reactions such as pain in extremity, infusion‐site pain, or mild phlebitis, but these effects may be able to be mitigated in future studies by altering the method of intravenous drug delivery. Second, the PK analyses of MW189 showed approximately linear kinetics, dose proportionality, no significant accumulation after multiple doses, a sufficiently long half‐life for twice‐daily dosing, and steady state achieved by dose 3 for all dosing cohorts. Third, the endotoxin study results provide initial evidence of a PD effect in humans at the dose proposed for phase 2a studies (0.25 mg/kg), consistent with the calculations that this dose would achieve comparable exposures associated with efficacy in animal models. Overall, these studies support further development of MW189 for treatment of patients with acute brain injuries such as TBI or hemorrhagic stroke.

## Conflicts of Interest

L.V.E. and D.M.W. are inventors on the patents covering MW189. L.V.E. is a scientific founder of ImmunoChem Therapeutics, LLC, a Northwestern spin‐out formed to commercialize MW189. Northwestern University and the University of Kentucky might benefit if MW189 is successful commercially. J.T.G. is supported by the National Institute of Neurological Disorders and Stroke of the National Institutes of Health under Award Number K23NS085049.

## Funding

This study was supported by an Alzheimer's Association grant PTC‐17‐445974.

## Data‐Sharing Statement

Requests for data‐sharing should be addressed to the corresponding author.

## Supporting information

Supporting Information
