# Supplementary material for: First‐in‐Human Studies of MW01‐6‐189WH, a Brain‐Penetrant, Antineuroinflammatory Small‐Molecule Drug Candidate: Phase 1 Safety, Tolerability, Pharmacokinetic, and Pharmacodynamic Studies in Healthy Adult Volunteers
Source: Clin Pharmacol Drug Dev. 2020 Apr 7;10(2):131–43. doi: 10.1002/cpdd.795 (PMC7541708; doi:10.1002/cpdd.795)
Supplement: Supplementary file 1 — Supporting Information [file CPDD-10-131-s001.docx]

**SUPPLEMENTARY MATERIAL**

First-in-human studies of MW01-6-189WH, a brain-penetrant, anti-neuroinflammatory, small molecule drug candidate: phase 1 safety, tolerability, pharmacokinetic, and pharmacodynamic studies in healthy adult volunteers

Linda J. Van Eldik et al.

**Supplemental Table S1**

**MW189 Properties**

| **Chemistry** | **Value/Properties** |
| --- | --- |
| Molecular weight (CAS # 886208-76-0) | 395.47 |
| cLogP (ACD v18) | 3.28 |
| Polar surface area | 70.93 |
| pKa (base) | 3.71 ± 0.09 (potentiometric) |
| Solubility of API (CAS # 1352919-06-02) | >60 mg/ml in water |
| Chemical stability GMP API (lot 1004878):  6-month time point  Accelerated study at 25°C ± 2°C; 60% RH ± 5% | 99.5% purity by HPLC  All tests within acceptance criteria |
| Chemical stability GMP API (lot 1004878):  24-month time point  Long-term study at 5°C ± 3°C; RH not controlled | 94.6% purity by HPLC  All tests within acceptance criteria |
|  |  |
| **Metabolic Stability and Distribution** | **Value/Properties** |
| *In vitro* metabolite profiling in primary hepatocytes from rat, dog, human:  10 μM MW189 for 180 min | 25-70% decrease over 180 min; oxidative (hydroxylation) metabolites most prominent, with smaller amounts of secondary phase II metabolites (sulfation and glucuronidation). No metabolites present in humans that were not present in either rat or dog |
| *In vitro* cytochrome P450 inhibition in human liver microsomes; MW189 0.01 μM to 25 μM | CYP Inhibition: negative for 2D6, 2E1, 3A4/5; 30% inhibition at 25 μM for 1A2, 2B6, 2C19 2C8; positive for 2C9 (IC_50_=10.3 μM) |
| *In vitro* plasma protein binding in human plasma | Stable in plasma for >5h at 37°C. 98-99% plasma protein binding |
| CSF/plasma exposure in dogs, based on AUC_0-inf_ | 5.81% at 3.75 mg/kg; 8.38% at 0.75 mg/kg |
| Oral bioavailability | 9% in rat; 24-28% in dog |
|  |  |
| **Adverse Pharmacology and Toxicology** | **Value/Properties** |
| *In vitro* GPCR binding screen at 10 μM MW189; 55 receptors, ion channels, transporters | All negative except Adenosine A-1 receptor (IC_50_=4 μM). Follow-up analysis showed no functional antagonist or agonist activity |
| *In vitro* kinase inhibitor screen at 10 μM MW189;  412 protein and lipid kinases | All negative (Roy et al., 2019, J Med Chem 62:5298-5311) |
| Genotoxicity: chromosomal aberration, mouse lymphoma, bacterial mutation, rat micronucleus | All negative |
| Rat CNS, dog cardiovascular, dog respiratory | Negative at highest doses tested: 7.5 mg/kg *iv* rats, 3.75 mg/kg *iv* dogs |
| 14d *iv* tox in rats at 3, 7.5, 15 mg/kg *bid*, with 14d recovery | No adverse events; drug well tolerated; NOAEL = 15 mg/kg  (highest dose tested) |
| 14d *iv* tox in dogs at 1.5, 3.5, 7.5 mg/kg *bid*, with 14d recovery | No adverse events; drug well tolerated; NOAEL = 7.5 mg/kg  (highest dose tested) |
| 28d oral tox in rats at 10, 30, 100 mg/kg daily, with 4-week recovery | No adverse events; drug well tolerated; NOAEL = 100 mg/kg  (highest dose tested) |
| 28d oral tox in dogs at 5, 15, 50 mg/kg daily, with 4-week recovery | No adverse events; drug well tolerated; NOAEL = 50 mg/kg  (highest dose tested) |

**Supplemental Table S2**

**Dose at which MW189 Steady State was Achieved**

| **Dose** | **Contrast** | **p-value** | | | |
| --- | --- | --- | --- | --- | --- |
|  |  | **0.075 mg/kg** | **0.15 mg/kg** | **0.25 mg/kg** | **0.30 mg/kg** |
| 1 | 1 vs 2, 3, 5, 7, 9 | 0.0083 | 0.0040 | 0.0015 | 0.0006 |
| 2 | 2 vs 3, 5, 7, 9 | **0.4800** | **0.1498** | 0.0354 | 0.0083 |
| 3 | 3 vs 5, 7, 9 | 0.9104 | 0.8530 | **0.7729** | **0.3183** |
| 5 | 5 vs 7, 9 | 0.8269 | 0.6389 | 0.5606 | 0.5976 |
| 7 | 7 vs 9 | 0.3276 | 0.4580 | 0.3094 | 0.0342 |

Steady state was assumed when trough concentration was not significantly different from subsequent trough concentrations (**bolded**). Steady state was achieved following the second dose in the 0.075 mg/kg and 0.15 mg/kg MW189 cohorts based on non-significant difference in C_min_ values for subsequent doses. Steady state was achieved after the third dose in the 0.25 mg/kg and 0.30 mg/kg MW189 cohorts.

**Supplemental Table S3**

**Response by time summary of MW189 versus placebo effects on LPS-induced cytokine levels**

| **Cytokine** | **Treatment by Time**  **(Pr > F)** |  | **Treatment by Time (Pr > F)**  **Mean plasma cytokine levels at individual time points**  (only cytokines for which a significant treatment by time interaction was detected are displayed) | | | | | | | | | |
| --- | --- | --- | --- | --- | --- | --- | --- | --- | --- | --- | --- | --- |
|  | **Mean: all time pts*** |  | **0h** | **0.5h** | **1h** | **1.5h** | **2h** | **3h** | **4h** | **6h** | **8h** | **12h** |
| IL-1ra | 0.131 |  | − | − | − | − | − | − | − | − | − | − |
| IL-6 | 0.981 |  | − | − | − | − | − | − | − | − | − | − |
| IL-8 | 0.314 |  | − | − | − | − | − | − | − | − | − | − |
| IL-10 | 0.010 * |  | − | − | 0.0070 | − | 0.0190 | 0.0012 | 0.0018 | − | − | − |
| TNFα | 0.005 * |  | − | − | − | − | − | − | − | 0.0004 | 0.0099 | 0.0002 |
| CCL2 | 0.833 |  | − | − | − | − | − | − | − | − | − | − |

*Mean plasma levels for each cytokine over the 12h time period following LPS were compared using ANOVA for MW189 versus placebo. Statistically significant Treatment by Time effects were observed for IL-10 and TNFα. Further comparison of mean cytokine levels at individual time points showed that the MW189 group showed significantly higher levels of IL-10 from 1-4 hrs post LPS infusion, and significantly lower levels of TNFα from 6-12h post LPS infusion compared to the placebo group.

“−” = not significant. Pr>F = p-value associated with the F statistic for Treatment by Time effects for mean values of MW189-treated versus placebo-treated groups.
